# Patient-specific variability in breath-hold positions during cardiac magnetic resonance imaging has a negligible effect on measures of cardiac mechanics

**DOI:** 10.1186/1532-429X-17-S1-P78

**Published:** 2015-02-03

**Authors:** Sean M Hamlet, Kristin Andres, Gregory J Wehner, Jonathan D Suever, David Powell, Xiaodong Zhong, Frederick H Epstein, Brandon K Fornwalt

**Affiliations:** 1 University of Kentucky, Lexington, KY, USA; 2 MR R&D Collaborations, Siemens Healthcare, Atlanta, GA, USA; 3 Biomedical Engineering, University of Virginia, Charlottesville, VA, USA

## Background

Cardiac magnetic resonance (CMR) can be used to quantify cardiac mechanics from images that are generally acquired during an end-expiratory breath-hold. Unfortunately, it is difficult for subjects to hold their breath at the exact same position when undergoing a series of breath-holds during a typical CMR study. The effect of patient-specific variability in breath-hold positions on measures of cardiac mechanics has not been investigated. We *hypothesized* that normal variability in breath-hold positions would significantly affect estimates of left ventricular strains and torsion.

## Methods

Ten healthy volunteers (Age: 29±10 years, 60% female) and 6 patients with a history of cardiovascular disease or myocardial infarction (Age: 58±9 years, 50% female) were consented. A 3T Siemens Tim Trio was used to measure the diaphragm position during ten 10-second breath-holds to determine each subject's breath-hold range. The diaphragm was measured at a sampling rate of 3Hz with the respiratory navigator sequence built into the DENSE sequence. A navigator feedback system enabled subjects to view their diaphragm position in real-time during image acquisition by using an angled mirror and projector screen placed at the back of the scanner bore. We acquired navigator-gated 2-chamber, 4-chamber, basal, mid-ventricular, and apical slices of 2D cine DENSE at the subject-specific maximum, middle and minimum breath-hold positions (with repeated middle position for inter-test quantification). The 2D DENSE parameters were: 6 spiral interleaves, voxel size = 2.8x2.8x8 mm, TE/TR = 1.08/17, flip angle = variable 20°, navigator acceptance window = ±3mm. Radial, circumferential, and longitudinal strains and torsion were calculated for each subject and compared between diaphragm locations using a repeated measures ANOVA. The inter-test 95% limits of agreement were calculated for strains and torsion using the Bland-Altman method. Since torsion is quantified from three independent short-axis images acquired during separate breath-holds, and we acquired each short-axis image at 3 different breath-hold locations, torsion was computed from each of 27 possible slice combinations (9 unique combinations).

## Results

**Figure 1:**
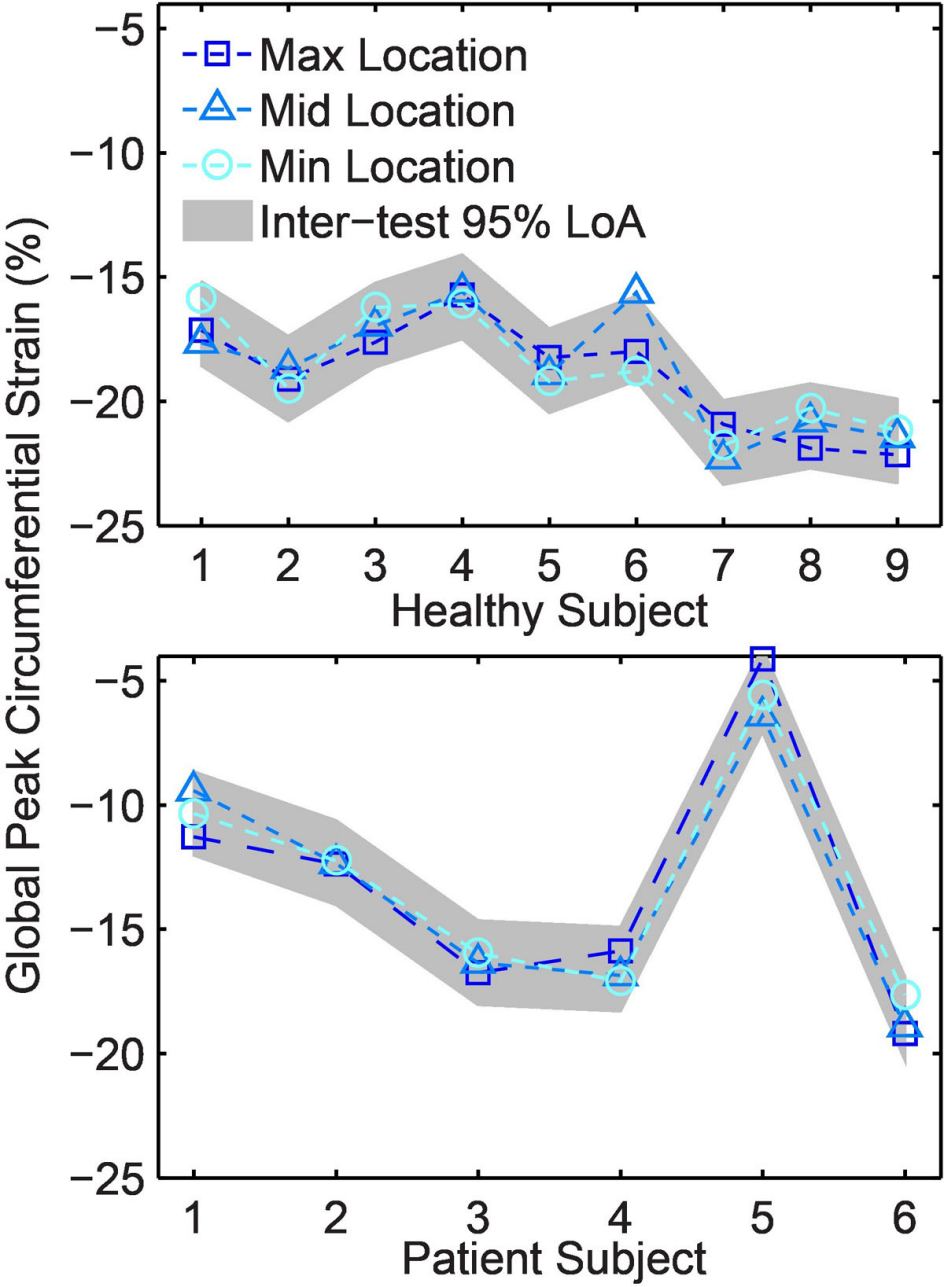
The differences for global peak circumferential left ventricular strain between the 3 different patient-specific breath-hold positions (minimum, middle, and maximum) were not significant and smaller than the inter-test 95% limits of agreement (LoA) for each subject group (shown in gray).

**Figure 2:**
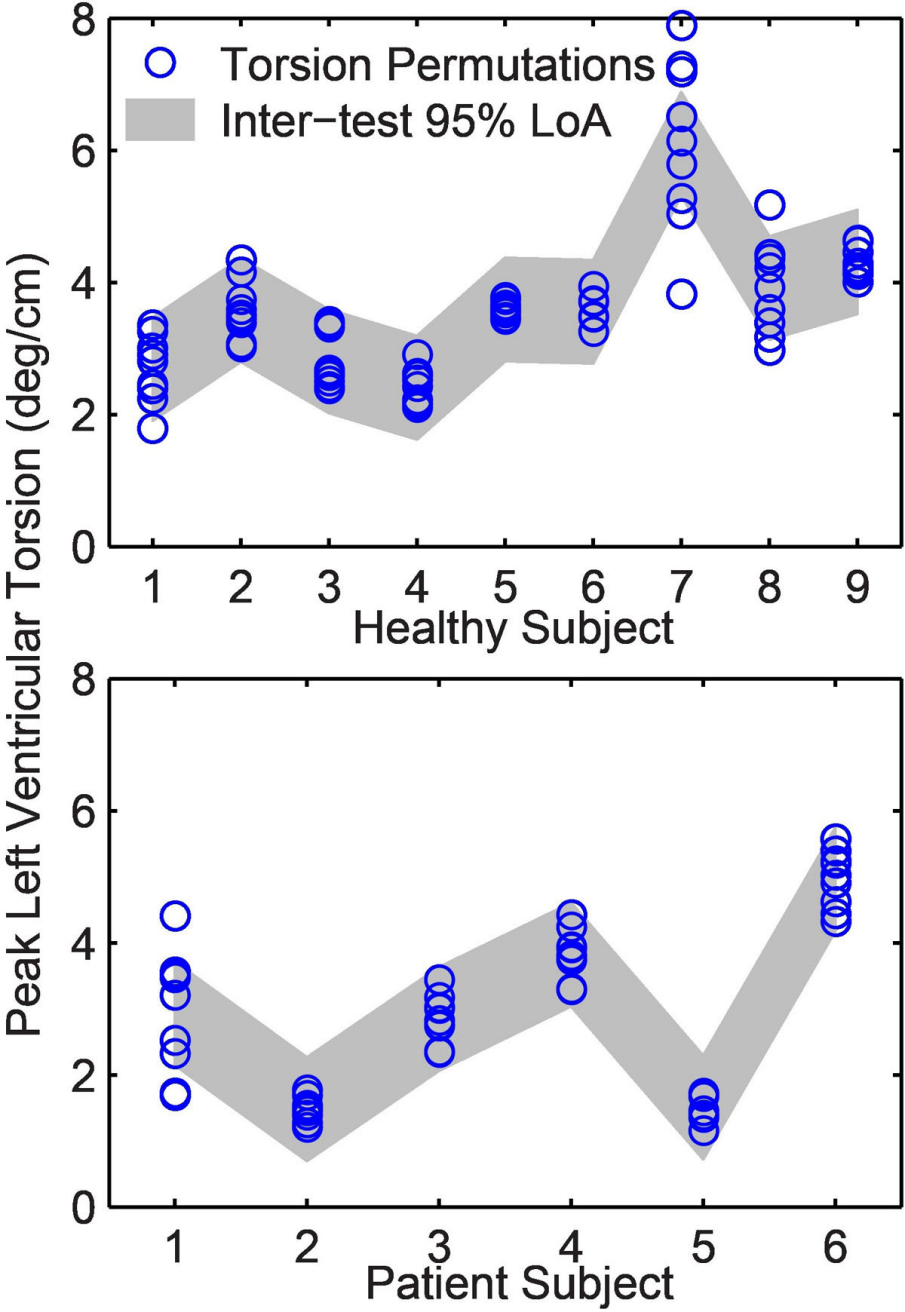
Most of the permutations of peak torsion for each subject fell within the torsion inter-test 95% limits of agreement (LoA) for each subject group (shown in gray).

One healthy subject was excluded due to patient movement. The average breath-hold range was 10 mm for both the healthy and patient groups. The breath-hold range varied from 4 to 19 mm for both groups combined. There were no clear trends or significant differences in strains and torsion measured at the different diaphragm positions. Furthermore, the differences were smaller than the inter-test 95% limits of agreement (Fig 1). Most torsion permutations fell within the inter-test 95% limits of agreement, suggesting that diaphragm position has minimal effect on the calculation of torsion (Fig 2).

## Conclusions

Different patient-specific breath-hold positions had no significant effect on the quantification of peak left ventricular cardiac strains and torsion from two-dimensional DENSE CMR.

## Funding

This work was supported by a National Institutes of Health (NIH) Director's Early Independence Award (DP5 OD-012132); the University of Kentucky Cardiovascular Research Center, grant UL1RR033173 from the National Center for Research Resources (NCRR), funded by the Office of the Director, National Institutes of Health (NIH) and supported by the NIH Roadmap for Medical Research. The content is solely the responsibility of the authors and does not necessarily represent the official views of NIH.

